# The incidence of consecutive manifestations in Von Hippel-Lindau disease

**DOI:** 10.1007/s10689-019-00131-x

**Published:** 2019-05-13

**Authors:** Anouk N. A. van der Horst-Schrivers, Wim J. Sluiter, Roeliene C. Kruizinga, Rachel S. van Leeuwaarde, Rachel Giles, Maran J. W. Olderode-Berends, Thera P. Links

**Affiliations:** 1 Department of Endocrinology, University Medical Center Groningen, University of Groningen, Groningen, The Netherlands; 2 Department of Medical Genetics, University Medical Center Groningen, University Medical Center Groningen, P.O. Box 30.001, 9700 RB Groningen, The Netherlands; 3 Department of Geriatric Medicine, Amsterdam UMC, Free University Amsterdam, Amsterdam, The Netherlands; 4 Department of Endocrine Oncology, University Medical Center Utrecht, Utrecht, The Netherlands; 5 Department of Nephrology, University Medical Center Utrecht, Utrecht, The Netherlands

**Keywords:** Von Hippel-Lindau disease, VHL-related manifestations, Disease progression, Surveillance

## Abstract

Von Hippel-Lindau (VHL) disease is an autosomal dominant rare tumor syndrome characterized by high penetrance. *VHL* mutation carriers develop numerous manifestations in multiple organs during life. The natural course of development of new and growth of existing VHL-related manifestations is still unclear. In this study we aimed to gain insight into the development of subsequent manifestations in VHL disease. We retrospectively scored each new VHL-related manifestation as detected by standard follow-up (retina, central nervous system, kidneys and pancreas, excluding adrenal and endolymfatic sac manifestations) in 75 *VHL* mutation carriers. The Kaplan–Meier method was used to plot the cumulative proportions of all consecutive manifestations in each organ against age. The cumulative average number of manifestations in all organs during life was calculated by summating these cumulative proportions. Poisson model parameters were used to calculate average time to the detection of consecutive VHL manifestations in each organ. Consecutive VHL-related kidney and retina manifestations during life occur linearly according to Poisson distribution model. The total number of VHL manifestations rises linearly, with an average of seven VHL-related lesions at age 60 years. The incidence of consecutive VHL-related manifestations is constant during life in *VHL* mutation carriers. Our data is consistent with the notion that somatic inactivation of the remaining allele (Knudson’s “two-hit” hypothesis) is the determining factor in developing new VHL-related manifestations.

**Electronic supplementary material:**

The online version of this article (10.1007/s10689-019-00131-x) contains supplementary material, which is available to authorized users.

## Introduction

Von Hippel-Lindau disease (VHL) is a rare inherited tumor syndrome with an incidence of 1 in 39,000 to 1 in 91,000 and a penetrance of > 80% by age 60 [1, 2] and approaching 100% by age 75 [3, 4]. Affected individuals develop multiple VHL-related manifestations during life including hemangioblastoma in the central nervous system (CNS) and the retina, clear cell renal cell carcinomas (RCC), pheochromocytomas, neuroendocrine pancreatic tumors, and endolymphatic sac tumor. Cysts in the kidney and pancreas are also frequently found in VHL disease, whereas cystadenomas in the epididymis and broad ligament are less common [3, 4].

Growth of existing lesions and development of new manifestations both indicate progressive VHL disease. Regular surveillance of *VHL* mutation carriers to detect new or growing lesions consists of ophthalmologist consultation, imaging (Magnetic Resonance Imaging (MRI) or ultrasound) of CNS and abdomen, and urine and plasma biochemistry [3, 5]. The natural course of VHL- related manifestations is based on clinical observations and is still a matter of discussion; several pathophysiological and (epi)genetic factors are suggested to play a role. Two studies show heterogeneous tumor growth behavior in RCC, without correlation between initial tumor size and growth rate [6, 7]. Tumor formation and growth in RCC is influenced by the extracellular matrix, tumor vasculature and immune cells [8], but also secondary genetic events play a role [9]. Recently a high-resolution genome-wide view of chromosomal changes in sporadic hemangioblastoma identified 23 candidate genes beyond *VHL* for hemangioblastoma pathogenesis [10].

The characteristic “stuttered” growth (alternate slow/rapid growth) that is observed in CNS hemangioblastomas has not yet been explained [11, 12]. In addition, three case series generated contradicting findings regarding the influence of pregnancy on growth of CNS hemangioblastomas in *VHL* mutation carriers [13–15]. Interestingly, evaluation of CNS hemangioblastomas in VHL patients showed a faster growth rate of existing lesions in males, symptomatic tumors and tumors associated with cysts [16]. Lonser et al. also demonstrate an increased number of CNS tumors per year at a younger age (12–20 years). In contrast, another study found the highest rate of new CNS hemangioblastomas development at age 30–34 years [17]. The working hypothesis is that, as a classic tumor suppressor gene, *VHL* will acquire a somatic mutation in the wild-type allele, leading to early tumor development [18].

The way VHL disease progression is defined either growth of existing lesions or development of new manifestations, may be important in acquiring a better understanding of the natural course of the disease. The aim of this study is to gain insight into disease progression in VHL patients by focusing on organ-specific serial VHL-related manifestations. We evaluated the number of subsequent organ manifestations diagnosed during life and analyzed whether the increase of new manifestation is linear according to the Poisson model [19, 20], in accordance with Knudson’s “two-hit” hypothesis. If new subsequent VHL-related manifestations arise linearly according to the Poisson model, these new lesions should occur with a fixed mean interval per organ system during life in all VHL patients.

## Methods

### Patients

All *VHL* mutation carriers of 18 years or older who underwent surveillance according to Dutch VHL guidelines [21] between 1972 and 2012 in the VHL expertise centers University Medical Center Groningen (UMCG) and University Medical Center Utrecht (UMCU) were included. A *VHL* mutation carrier was defined as an individual with either a genetically confirmed *VHL* mutation or the presence of clinical VHL disease. A patient is clinically diagnosed with VHL when having two or more CNS hemangioblastomas, a typical CNS hemangioblastoma(s) and visceral lesion(s) or as one typical VHL-related manifestation and a first degree relative with genetically or clinically proven VHL disease [4]. Patients with VHL disease type 1 (including all typical VHL manifestations except pheochromcytoma) and type 2 A and B (exhibiting all typical VHL-related manifestations with low-2 A- and high-2B- risk for RCC) were included. Patients with VHL-disease subtype 2C (pheochromocytoma only) were excluded. The Dutch surveillance protocol consists of biannual MRI of the central nervous system (including spinal cord since 1991), annual ophthalmologist consultation and annual imaging of the abdomen; since 1985 annual abdominal ultrasound (at the UMCU and since 1990 at the UMCG) and since 2010 abdominal MRI biannual and if necessary abdominal ultrasound in alternate years. Intervals in surveillance were irrespective of detection of new VHL-related manifestations, but in case of new manifestations, lesion growth or the suspicion of malignancy the frequency was increased by repeating the procedure after 4–6 months and if stable after 1 year. Interim examinations or additional imaging was performed if the carrier developed symptoms. Annual plasma and/or urinary assessments of metanephrines were performed to detect a pheochromocytoma.

### Data collection procedures

Clinical data of 75 *VHL* mutation carriers were retrospectively retrieved from electronic and written patient records. Data were stored in an anonymous database with unique codes protecting patient identity. The codes were only held by three data managers. Therefore, according to Dutch law no further Institutional Review Board approval was required. Reports from the Department of Clinical Genetics were used for information on genealogy. If the diagnosis was based on clinical features preceding an imaging-based diagnosis, the first date of diagnosis was defined when the VHL lesion was confirmed by imaging.

### Detection of new VHL-related manifestations

For this study we evaluated retinal hemangioblastomas, CNS hemangioblastomas (cerebral and spinal cord), and kidney (complex cysts and RCC) and pancreatic lesions (including cysts or and neuroendocrine pancreatic tumors). New related VHL manifestations asymptomatic as well as symptomatic were defined as either a new retinal angioma reported by the ophthalmologist, a new hemangioblastoma larger than 1 cm reported in the CNS-MRI (cerebral and spinal cord), a new kidney lesion described in the report of the MRI or abdominal ultrasound (complex cysts and renal RCC) and a new pancreatic lesion reported in the MRI or abdominal ultrasound (including cysts and neuroendocrine pancreatic tumors or cystadenomas) regardless the symptoms. Hemangioblastomas smaller than 1 cm were excluded. All manifestations of hemangioblastoma after neurosurgery were included, for it was sometimes difficult to distinguish recurrent and new lesions. New renal tumors were scored after partial or total nephrectomy, however not used in the calculations, because the patients were censored after surgery, based on the expectable reduction of pick-up rate.Patients who underwent radical bilateral nephrectomy were excluded after the second nephrectomy. Pheochromocytomas were not included in this analysis because adrenalectomy prevented a second manifestation in the adrenal gland.

### Statistical analysis

The Poisson distribution expresses the probability of a given number of events occurring in a fixed interval of time or space assuming that the events occur with a known constant rate and independently of the time since the last event. In patients with VHL born with a germline mutation (i.e. the first hit) on chromosome 3, the ‘second’ hit resulting in a somatic mutation on the other, not affected gene on other chromosome 3 is the event that marks the start of an organ manifestation.

Poisson distribution model parameters per organ, based on the age of appearance of first organ-specific VHL-related manifestation, were calculated as described previously [18, 22]. Linear regression analysis of natural logarithm of 1-cumulative proportion (ln(1-cumulative proportion)) versus age was carried out to estimate the average incidence k (the organ specific hit rate) of tumor formation from the regression coefficient (= −k). The average time between two subsequent hits with its Standard Error (S.E.) was derived from the inverse of k and its S.E.). The average time between a first hit and detection of first VHL-related manifestation (delay) was assessed from the intercept of the ln(1-cumulative proportion) with the age axis. The S.E. of delay was defined by the S.E. around the regression line divided by the regression coefficient k. The median age of first manifestation was defined as the age at which half of the carriers experienced a first manifestation. Cumulative proportions below the 5% level were excluded from the regression analysis because of the sloping edge caused by the variance of delay [22]. The hit rate and delay between hit and appearance of a VHL manifestation for each organ were used to design Poisson distribution models predicting age of consecutive new manifestations. The mean time in years for an organ manifestation means that the first organ manifestation (after the first somatic mutation in that specific organ, i.e. the second hit) occurs on average that age of life (with of course a range of birth until death). The delay of an organ manifestation denotes the average time in years the tumor development takes before can be visualized by imaging. It must be realized that the moment of the detection by surveillance also has a window of 1 to 2 years based on the surveillance intervals.

For each organ the observed consecutive lesions were plotted against age in a Kaplan–Meier curve. The cumulative average number of VHL-related manifestations per organ is shown by the summation of these graphs. The cumulative average number of manifestations in all organs during life was calculated by summating the graphs of all organs.

## Results

### Patients

The characteristics of all 75 *VHL* mutation carriers and their manifestations are shown in Table 1. The median follow-up time was 19 years (range 1–41 years). Seven *VHL* mutation carriers died during follow-up; six because of VHL-related manifestations. The *VHL* gene mutations found in the study population covered all three exons of the *VHL* gene. A total of 342 lesions in the four organ systems (retina, CNS, kidney, pancreas) were included in this analysis (Supplementary Table 1).

**Table 1:** Characteristics of *VHL* mutation carriers

Characteristic	No. (%)	VHL-type	Mean age (years) at last FU with range	VHL-related manifestations
Sex				
Male	37 (49%)			
Female	38 (51%)			
Mutation				
c.208G > A	1 (1%)	1	20	HBr
c.-213-?_463 + ?del	4 (5%)	1	45 (38–48)	HBr, HBc, HBsc, RCC, PNET, Cr, Cp
c.241C > T	2 (3%)	2a/b	47 (34–59)	HBr, HBc,HBsc, RCC, PNET, Pheo, Cr
c.259_260-insA	1 (1%)	1	42	HBr, HBc,HBsc, RCC, Cr, Cp
c.277G > A	2 (3%)	2a/b	48 (35–60)	HBr, HBsc, Pheo, Cr, Cp
c. 89_297del	25 (33%)	1	40 (17–70)	HBr, HBc,HBsc, RCC, PNET, Cr, Cp
c.340 + 1G > A	1 (1%)	1	32	HBr, HBc, HBsc, RCC, Cr, Cp
c.341-59_341-14del	2 (3%)	2a/b	52 (33–67)	HBr, HBc, RCC, Pheo, Cr, Cp
c.358A > G	1 (1%)	1	15	HBsc
c.407T > C	1 (1%)	1	41	HBc, HBsc, RCC, Cr, Cp
c.462A > C	1 (1%)	2a/b	71	HBc, Pheo. Cr
c.497T > C	1 (1%)	2a/b	27	PNET, Pheo
c.499C > T	1 (1%)	2a/b	43	HBr, HBsc, RCC, Pheo, Cr
c.500G > A	11 (15%)	2a/b	45 (21–65)	HBr, HBc,HBsc, RCC,pNET, Pheo, Cr, Cp
c.509T > A	15 (20%)	2a/b	45 (16–71)	HBr, HBc,HBsc, RCC, pNET, Pheo, Cr, Cp
c.565delG	1 (1%)	1	30	HBc, Cr
c.463 + 2T > C p.(?)	1 (1%)	1	31	HBr, HBc, HBsc, RCC, Cr
c.464-3C > T	1 (1%)	1	63	HBr, HBc, HBsc, RCC, Cr
Rearrangement SB	1 (1%)	1	25	HBr, HBc, HBsc, Cr, Cp
Unknown	1 (1%)	1	44	HBr, HBc, HBsc, RCC, Cr, Cp
Nothing found	1 (1%)	1	40	HBr, HBc, Cr
VHL-phenotype:				
Phenotype 1	40 (53%)			
Phenotype 2a/b	35 (47%)			
Mean FU in years			19 (0–45)	

*SB* Southern blot removes exon 1 no further details available, *HBr* retinal hemangioblastoma, *HBc* cerebellar hemangioblastoma, *HBsc* spinal cord hemangioblastoma, *RCC* renal cell cancer, *pNET* pancreatic neuroendocrine tumor, *Pheo*, pheochromocytoma, *Cr* renal cyst, *Cp* pancreatic cyst, *FU* follow up

### Incidence of consecutive VHL-related manifestations per organ

#### Retinal manifestations

**Fig. 1:**
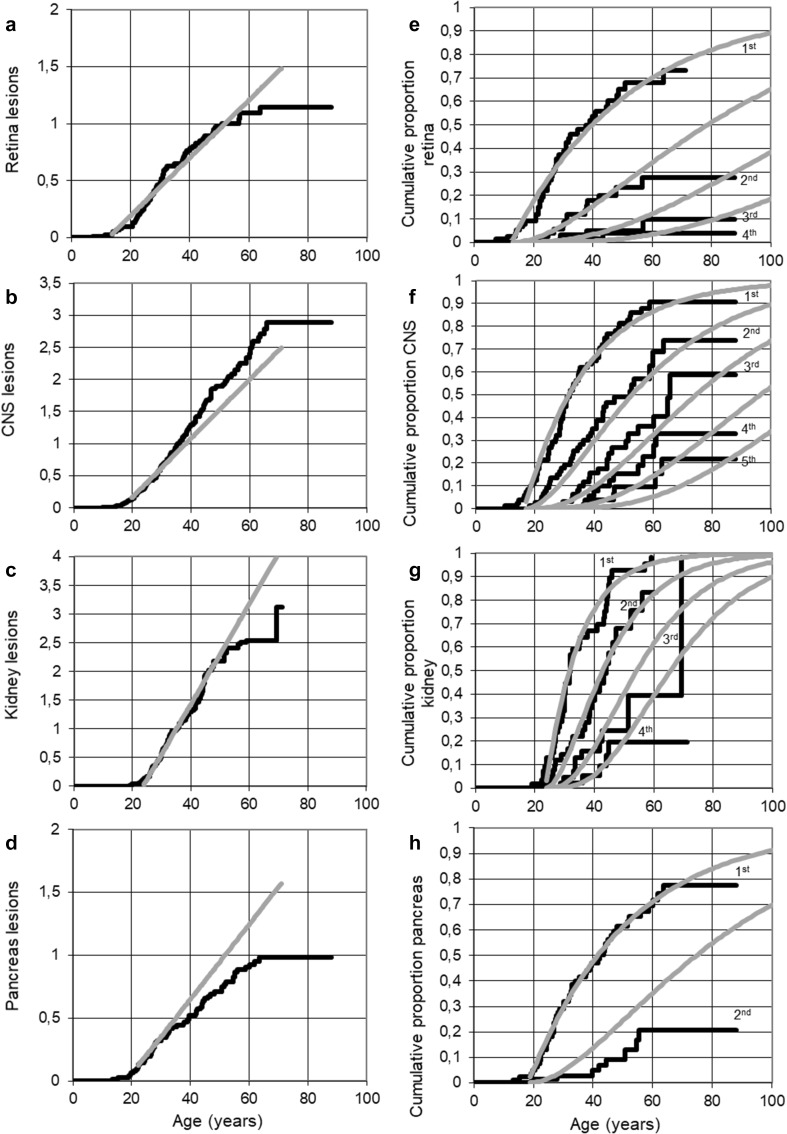
Predicted cumulative proportions of consecutive VHL-related manifestations for each organ (**a**, **b**, **c**, **d**) and Lifelong cumulative lesions in VHL mutations carriers during life (**e**, **f**, **g**, **h**) using the Poisson model (grey lines) compared to observed lesion counts during life (black lines) in retina, CNS, kidney and pancreas

The average time between two subsequent hits for the retina was 39.4 years, with a delay after the second hit of 12.4 years (S.E. 2.83); age at median first manifestation was 39.9 years (Table 2). About 75% of *VHL* mutation carriers were diagnosed with at least one retinal angioma and 10% with at least 3 retinal angiomas (Fig. 1a). The increase in number of retinal angiomas was linear, with an average of 1 retinal angioma per *VHL* carrier at age 50 (Fig. 1b).

**Table 2:** Poisson model parameters in *VHL* mutation carriers

	TBH (years)	Delay (years)	SE (years)	AMFM (years)
Retina	39.4	12.4	2.83	39.9
CNS	21.1	18.1	2.25	31.3
Kidney	11.4	23.6	2.71	31.5
Pancreas	33.6	18.2	1.2	41.7

*CNS* central nervous system including cerebellum and spinal cord, *TBH* time between hits; Delay, delay between second hit and manifestation, *AMF* age at median first manifestation

#### CNS manifestations

The average time between two subsequent hits resulting in CNS hemangioblastoma was 21.1 years, with a delay of 18.1 years (S.E. 2.25); age at median first manifestation was 31.3 years (Table 2). Almost every *VHL* mutation carrier was diagnosed with at least one CNS hemangioblastoma and 20% of the patients 5 or more lesions (Fig. 1a). Observed consecutive CNS hemangioblastomas arose progressively earlier than predicted by the Poisson model, starting around age 20. The apparent incidence of new CNS manifestations increased with 15% after the first lesion was detected. By the age of 50, an average of two CNS hemangioblastomas were detected (Fig. 1b).

Hemangioblastomas in the cerebellum and spinal cord were analyzed together as one organ system, defined as CNS hemangioblastomas. Therefore, the hit rate and delay in this study are slightly different from the earlier reported parameters (Kruizinga 2013).

#### Kidney manifestations

The average time between two subsequent hits of the kidney was 11.4 years, with a delay of 23.6 years (S.E. 2.71); age at median first manifestation was 31.5 years (Table 2).

Almost all *VHL* mutation carriers were diagnosed with at least one VHL-related kidney manifestation and 40% of all VHL patients develop at least 3 kidney lesions (Fig. 1a). By the age of 50, VHL patients develop an average of 2 kidney manifestations (Fig. 1b). The increase in the number of kidney lesions was linear up to 50 years, but at higher ages, was lower than expected.

#### Pancreas manifestations

The average time between two subsequent hits for the pancreas was 11.4 years, with a delay of 18.2 years (S.E. 1.2); age at median first manifestation was 41.7 years (Table 2). The proportion of VHL patients diagnosed with a second pancreatic lesion was about 50% of the predicted proportion by Poisson (Fig. 1a). 80% of all *VHL* mutation carriers will be diagnosed with 1 pancreatic manifestation during their life and 20% with 2 lesions. The increase in number of pancreatic manifestations is linear at first, as predicted, but declined as age advanced (Fig. 1b).

### Cumulative number of VHL-related manifestations during life

**Fig. 2:**
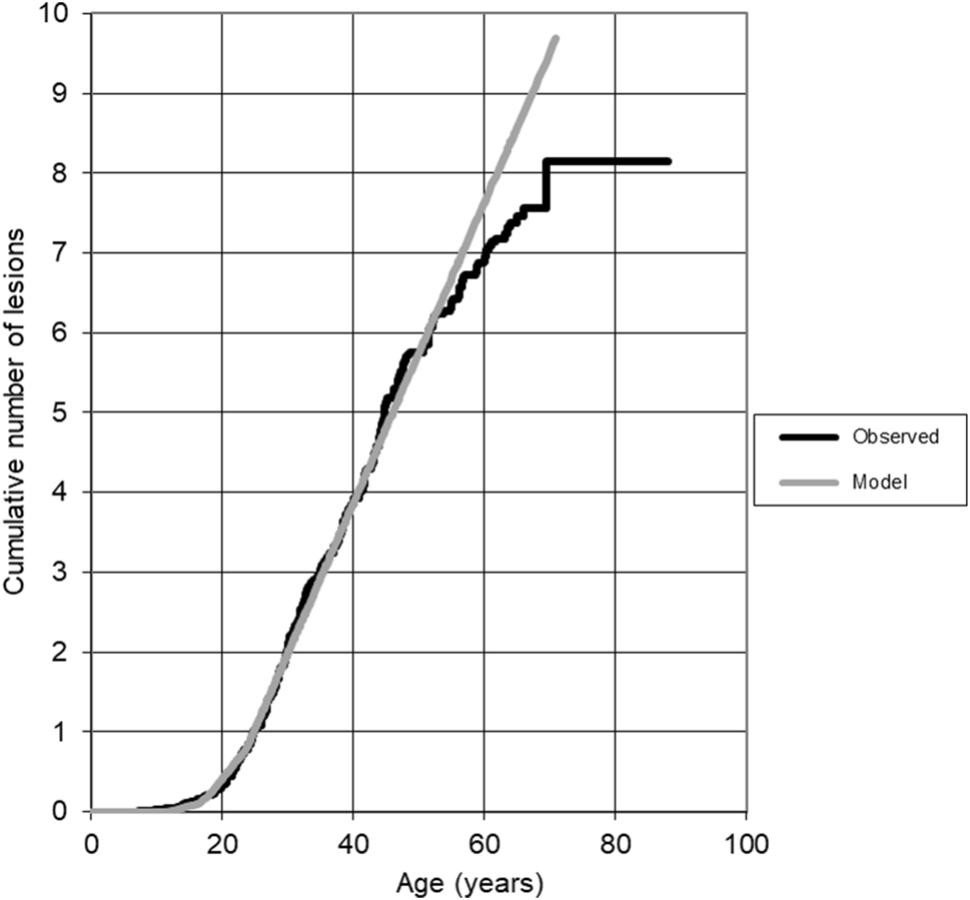
Average cumulative number of VHL-related manifestation during life in VHL mutation carriers summated for all organs; Poisson model (grey line) versus observed data (black line)

The increase in predicted and observed cumulative average numbers of all VHL manifestations is shown in Fig. 2. Until the age of 60 years the increase is as predicted by the Poisson model. After 60 years the predicted number of lesions were slightly higher than the number reported. *VHL* mutation carriers developed an average of seven VHL lesions by the age of 60.

## Discussion

In this study we aimed to gain insight into VHL disease activity with regard to the incidence of new organ-specific manifestations regardless if these lesions were symptomatic or asymptomatic. Using the Poisson model parameters derived from the observed data of first manifestation in each specific organ, predictions of age at diagnosis of new or consecutive VHL-related manifestations in these organs were made, as well as the total average number of manifestations per carrier during life. These predictions were compared with the clinical data in the records of all included VHL patients.

We conclude that consecutive VHL-related kidney and retina manifestations during life occur linearly according to the Poisson distribution model. Consecutive CNS hemangioblastomas were detected earlier than predicted according to the Poisson distribution and the second pancreatic lesion occurred later than predicted by the Poisson model. The total systemic number of VHL manifestations rises linearly, with an average of seven VHL-related manifestations at age 60 years. Our findings confirm previous knowledge that surveillance for VHL disease should be lifelong. In addition we show that the occurrence of consecutive of VHL manifestations occurs linear. So, a short interval between manifestations in a patient does not implicate a different, more aggressive change of VHL disease.

It should be emphasized that this study is retrospective and based on the report of events in patient records. Thus, for CNS manifestations for example, we only accounted for the reported incidence of indication for neurosurgical intervention of lesions larger than 1 cm. As such, we did not differentiate whether the lesion was new or recurrent and we ignored smaller lesions that did not have indication for intervention. Likewise, the records of pancreatic lesions in many cases reported only the presence or absence of cysts, as the number of observed lesions was often not reported due to its limited relevance in daily practice.

Observed manifestations in the kidney and the retina arose linearly with age after an organ-specific delay as expected from the Poisson model. These real-world data support the notion that consecutive manifestations occur with fixed intervals without any age-related variance until the age of 60, after which mortality progressively reduces the observed incidence to below model-expected values. Similarly, linear relation between age and indication for surgery was also observed in CNS lesions. However, new lesions were observed about 15% more frequently than predicted, suggesting that about 15% of second and consecutive indications for surgery were likely recurrences of incompletely resected prior lesions, typically observed within a year after treatment. In contrast, the expected linear relation between age and manifestations in the pancreas was only present for the first manifestation. This incongruity might be attributable to the fact that only about half of the records accurately reported the number and nature of additional lesions, whereas the other half only indicated the presence of unspecified cysts.

At first glance, our results seem to show discrepancies with the Danish VHL study [17] and the American CNS study [16]. Binderup et al. showed that the rate of new tumor formation was highest for retinal lesions in teenage years and was highest for cerebellar hemangioblastomas during the fourth decade of life. The rate of new tumor development for all VHL manifestations was highest at age 30–34 years with 0.4 new lesions per year. De facto these data are very similar to our results; the delay for retinal lesions to become manifest reaches to the early teenage years while for CNS lesions this delay is somewhat longer, whereas the maximum rate for all manifestations is reached only after the first manifestation of kidney lesions at about 25 years of age. Lonser et al. [16] reported not only on CNS lesions with indication for surgery, but also much smaller ones. They observed the increase in CNS manifestations between 12 and 20 years of age, indicating that the delay of detection of small lesions is, expectably, shorter than for the larger ones needing surgery. Notably, Lonser et al. also show that most of the smaller lesions never reach the size for indicated intervention and thus the rate of formation of CNS lesions is much higher in their study.

According to our results new VHL manifestations develop at a constant rate per organ system during life and are therefore the result of random somatic genetic insults. Over 1850 germline and somatic mutations are published worldwide in the *VHL* gene located in the p25 region of chromosome 3 [23, 24]. According to Knudson’s two-hit hypothesis, the start of tumorgenesis occurs as a result of biallelic loss of a tumor suppressor gene [19] and confirmed by Maher [25], who showed that both cerebellar hemangioblastoma and renal cell carcinoma in VHL disease followed the same mutation model. Although biallelic *VHL* inactivation and the resulting activation of HIF and its targets seem to be necessary for tumor development, other activating factors are also needed for initiation of tumor progression [19]. Loss of the *VHL* wild-type allele results from deletions or point mutations [26–28], although mutations seem not to be restricted to the 3p25.3 region [9]. On the other hand, growth of existing VHL lesions seems even more complex. The increase in size of the solid and/or cystic components may be the result of several local pathophysiological and (epi)genetic factors over time.

Although not fully understood, Nordstrom [29] described the spectrum of mutation types and the clinical spectrum, without the understanding how VHL mutations influence phenotypes. However, these data may be supportive for us to be capable to identify VHL mutations with the intent to inform the patient about possible outcome and to modify and optimize therapeutic regimen.

A limitation of our study is the retrospective design where we were unable to evaluate growth of individual lesions over time. Therefore we only studied new manifestations. This limitations resulted in the discrepancy between the observed number of pancreas and CNS lesions compared to the Poisson distribution, based on the underreporting of pancreas cysts and the difficulty in discrimination between a new or a recurrent lesion. Furthermore, there was systematic underestimation of new kidney lesions > 50 years age, for two reasons. Firstly, no adjustment was made for partial or unilateral nephrectomy in some patients during follow-up. And secondly, in some patients existing cysts increased in volume to such an extent, that it became virtually impossible to recognize any new lesions, reported as “complex cysts”. Manifestations in the retina as observed arose linearly with age after the organ-specific delay, as expected from the Poisson model. We conclude that consecutive manifestations occurred with fixed intervals without any age-related variance until the age of 60, after which mortality progressively reduces the observed incidence to below model-expected values. Kidney manifestations were also linear up to 50 years, but thereafter dropped to fewer than expected due to partial and radical nephrectomy, obfuscation of new lesions due to complex cysts, and age-related mortality.

Collectively, our data support continue standard surveillance of VHL mutation carriers lifelong to find new manifestations. To date, we have very little data regarding the extent and manner in which environmental and (epi)genetic factors contribute to the growth of an existing lesion; research in these areas will lend novel insights into the natural course of disease in VHL. Lastly, because fast growing lesions may give rise to symptoms, loss of function and the need for immediate intervention, we encourage all VHL health care providers to uniformly report the development of new lesions and growth of existing lesions in patient’s medical records.

## Electronic supplementary material

Below is the link to the electronic supplementary material.

Supplementary material 1 (DOCX 14 kb)

Supplementary material 2 (XLSX 23 kb)
