# Supplementary material for: The incidence of consecutive manifestations in Von Hippel-Lindau disease
Source: Fam Cancer. 2019 May 13;18(3):369–76. doi: 10.1007/s10689-019-00131-x (PMC6560011; doi:10.1007/s10689-019-00131-x)
Supplement: Supplementary file 1 — Supplementary material 1 (DOCX 14 kb) [file 10689_2019_131_MOESM1_ESM.docx]

**TABLE S**

Number of evaluated lesions per organ system

|  | 1^st^ lesion | 2^nd^ lesion | 3^rd^ lesion | 4^th^ lesion | 5^th^ lesion |
| --- | --- | --- | --- | --- | --- |
| Retina | 42 | 14 | 4 | 2 |  |
| CNS | 65 | 39 | 20 | 11 | 6 |
| Kidney | 41 | 24 | 10 | 5 |  |
| Pancreas | 51 | 8 |  |  |  |
